# Sequential severe immune-related adverse events induced by PD-1 inhibitor: a case report and literature review

**DOI:** 10.3389/fonc.2024.1391698

**Published:** 2024-07-29

**Authors:** Jinxiong Xia, Yingmei Wen, Mengxia Xiao, Dafu Ye, Yanjun Gao, Dongling Tang, Xiuyun Zhang, Jinling Chen, Qingqing Li, Yi Yao

**Affiliations:** ^1^ Cancer Center, Renmin Hospital of Wuhan University, Wuhan, China; ^2^ Department of Oncology, Yichun People’s Hospital, Yichun, China; ^3^ Department of Clinical Laboratory, Renmin Hospital of Wuhan University, Wuhan, China; ^4^ Department of Pathology, Renmin Hospital of Wuhan University, Wuhan, China; ^5^ Department of Ultrasound Imaging, Renmin Hospital of Wuhan University, Wuhan, China; ^6^ Hubei Provincial Research Center for Precision Medicine of Cancer, Wuhan, China

**Keywords:** advanced lung adenocarcinoma, sintilimab, immune-related adverse events, immune myocarditis, immune hepatitis, immune pneumonia, case report

## Abstract

In a variety of cancers, immune checkpoint inhibitors (ICIs) have demonstrated substantial survival advantages. Nevertheless, the widespread use of ICIs in the clinic has resulted in a growing interest in immune-related adverse events (irAEs) and their treatment methods. This paper reports a case in which a patient with three sequential severe irAEs was successfully treated. After undergoing two regimens of sintilimab in conjunction with chemotherapy for advanced lung cancer, the patient developed myocarditis combined with hepatitis. Subsequently, the patient developed pneumonia following remission from treatment. We also discuss the mechanism of irAEs, principles of treatment, and progress in the study of biomarkers for early prediction of irAEs by reviewing the literature.

## Introduction

Immune checkpoint inhibitors (ICIs) can be used to relieve immunosuppression by targeting and blocking the negative regulatory signals of T cells and promoting the recognition, killing, and apoptosis induction of cancer cells by CD8^+^ T cells. While ICIs improve anti-tumor immunity, they may also unnaturally augment the host’s autoimmune response, resulting in an imbalance of immunological tolerance and causing undesirable reactions to normal tissues, known as immune-related adverse events (irAEs) (1). The irAEs can develop at any stage of immunotherapy, and the majority of them are mild and reversible (2). Although rare, irAEs occurring in the myocardium, lung, brain, and liver tissues are frequently severe and even fatal. Here, we present the effective treatment of a patient with advanced lung cancer who developed immune-related myocarditis, hepatitis, and pneumonia sequentially following immunotherapy.

## Case presentation

**Figure 1:**
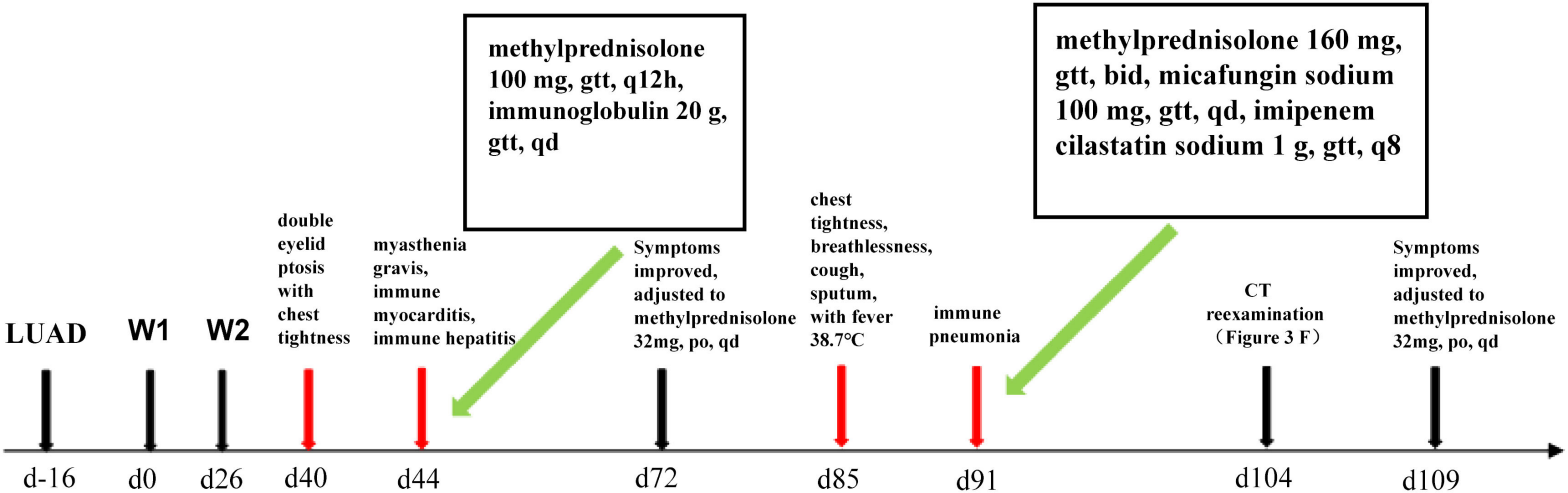
Timeline of immunized myocarditis and immunized pneumonia treated with corticosteroid interventions.

The patient, a 66-year-old male with stage IVB (cT3N2M1c, according to AJCC 8th edition) lung adenocarcinoma and metastasis to the left scapula, had lung tissue immunohistochemistry and NGS results showing a tumor PD-L1 TPS of 70% and no driver gene mutations, respectively. He had a history of coronary artery disease and unstable angina, but no history of liver disease. On 7 January 2021, he commenced treatment with sintilimab (200 mg day1) plus pemetrexed disodium 500 mg/m^2^ (day 1) and nedaplatin 80 mg/m^2^ (day 1 and day 2) every three weeks for two cycles at Renmin Hospital of Wuhan University. Concurrently, he was administered zoledronic acid (q.4w. 4 mg) treatment (schedule as in **Figure 1**).

**Figure 2:**
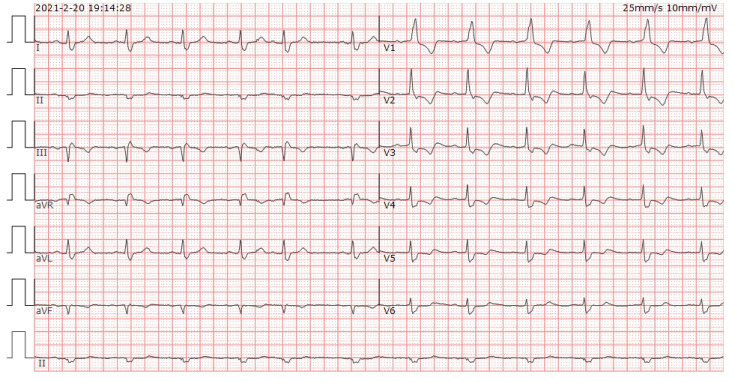
ECG of the patient at the onset of chest tightness. Heart rate was 71 beats/min. PR 192ms, QRS 147ms, OT 439ms, QTc 477ms. electrical axis: -63°. The diagnosis: sinus rhythm, severe left deviation of the electrical axis, complete right bundle branch block, left anterior branch block, and ST-T changes.

**Figure 3:**
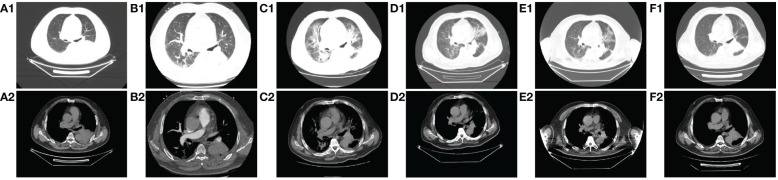
Comparison of CT images of patients before and after treatment with methylprednisolone and antibiotics. **A1-A2**: before immunotherapy (d-27); **B1-B2**: after 2 cycles of chemotherapy combined with immunotherapy (d45); **C1-C2**: immune pneumonitis before treatment with methylprednisolone and antibiotics (d88); **D1-D2**: day 4 after treatment of immune pneumonitis with methylprednisolone and antibiotics (d91); **E1-E2**: day 10 after treatment of immune pneumonia with methylprednisolone and antibiotics (d97); F1-F2: day 17 after treatment of immune pneumonia with methylprednisolone and antibiotics (d104).

On the 40th day, the patient exhibited double eyelid ptosis and chest tightness along with serum alanine aminotransferase (ALT) levels at 80.00 U/L (normal range, 9–50 U/L) and aspartate aminotransferase (AST) at 415.00 U/L (normal range, 15–40 U/L). The symptoms did not improve following the administration of oral pyridostigmine bromide (b.i.d. 120 mg) in the outpatient department. On day 44, the patient was admitted to the hospital. The blood tests showed: ALT 151.00 U/L, AST 769.00 U/L; the blood creatine kinase isoenzyme (CK-MB) 179.55 ng/mL (normal range, 0–5 ng/mL), myoglobin (MYO) >1000.00 ug/L (normal range, 0–110 ug/L), ultrasensitive troponin I (ultra-TNI) 4.396 ng/mL (normal range, 0–0.04 ng/mL), N-terminal pro-brain natriuretic peptide (NT-proBNP) 149.00 pg/mL (normal range, 0–125 pg/mL), blood interleukin (IL)-6 292.11 pg/mL (normal range, ≤20 pg/mL), IL-10 7.55 pg/mL (normal range, ≤5.9 pg/mL). The electrocardiogram showed sinus rhythm, severe left deviation of the heart’s electrical axis, complete right bundle-branch block, left anterior branch block, and ST-T changes (**Figure 2**). The echocardiogram demonstrated that the left atrium was enlarged, the overall systolic function of the left ventricle was satisfactory, and the left ventricular hypo diastolic function was grade 1. Coronary computed tomography angiography (CTA) indicated mild stenosis of the proximal and middle left anterior descending branch, as well as the proximal and middle right coronary artery lumen. The patient was unable to undergo cardiac magnetic resonance imaging (MRI) due to the severity of his condition. A comparison of the chest computed tomography (CT) images before and after treatment (**Figures 3A1-A2** vs **Figures 3B1-B2**) revealed that the patient achieved partial release (PR) according to the response evaluation criteria in solid tumors (RECIST1.1). In accordance with the *NCCN Guidelines for the Management of Immunotherapy-Related Toxicities, Version 1.2023*, the patient was diagnosed with irAEs: myocarditis (grade 3–4) in conjunction with hepatitis (grade 3). Immunotherapy and chemotherapy were suspended and intravenous methylprednisolone sodium succinate (3mg/kg/d) was initiated on day 45. Additionally, immunoglobulin (0.4g/kg/d for 3 days) was administered. After three days, the patient exhibited a notable improvement in ptosis, fatigue, chest tightness, and dyspnea. And the blood test results demonstrated a favorable shift: ALT 119.00 U/L, AST 254.00 U/L, CK-MB 45.70 ng/mL, MYO 986.69 ug/L, ultra-TNI 0.924 ng/mL, NT-proBNP 402.00 pg/mL, IL-6 4.81 pg/mL, IL-10 4.99 pg/mL. Following a 10-day course of treatment, the dose of methylprednisolone was reduced and replaced with tablets (32 mg/d) on day 72. This was followed by a further reduction to 16 mg once daily on day 80.

**Figure 4:**
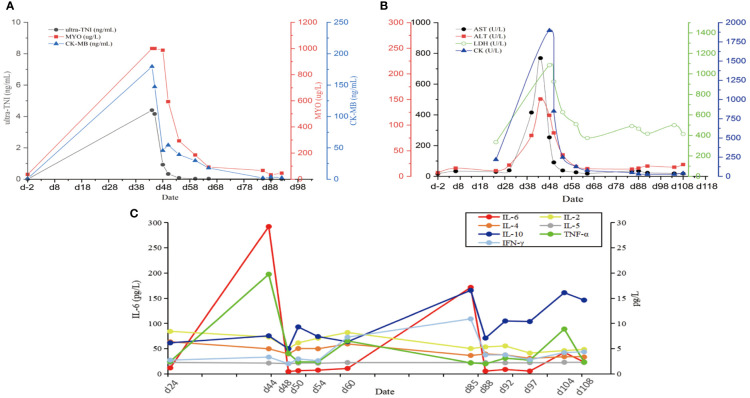
The changes of various blood indexes over time during continuous irAEs in patients. **(A)** Changes in blood markers of myocardial injury. **(B)** Changes in markers of liver function injury impairment. **(C)** Changes in cytokine levels. Ultra-TNI, Ultrasensitive Troponin I; MYO, Myoglobin; CK-MB, Creatine Kinase Isoenzyme; AST, Aspartate Aminotransferase; ALT, Alanine aminotransferase; LDH, Lactate dehydrogenase; CK, Creatine kinase; IL-2, Interleukin 2; IL-4, Interleukin 4; IL-6, Interleukin 6; IL-10, Interleukin 10; TNF-α, Tumor Necrosis Factor; INF-γ, Gamma-interferon.

On day 85, despite the continued administration of the oral dose of glucocorticoid (GCs) at 16 mg once daily, the patient once again exhibited signs of respiratory distress, including chest tightness, breathlessness, cough, and sputum production, accompanied by a fever (maximum axillary temperature of 38.7°C), and an ECOG PS score of 3. The blood neutrophil count was 7.85 × 10^9^ cells/L (normal range, 3.5–9.5 × 10^9^ cells/L), with an 86.80% percentage (normal range, 40%-75%). The procalcitonin (PCT) level was 0.320 ng/mL (normal range, ≤0.1 ng/mL), the IL-6 level was 171.55 pg/mL, the G test (fungal D-glucan) level was 153.55 pg/mL (normal range, ≤70 pg/mL), and the GM test was negative. Arterial blood gas analysis revealed a pH of 7.44 (normal range, 7.35–7.45), an oxygen partial pressure of 69 mmHg (normal range, 83–108 mmHg), a carbon dioxide partial pressure of 38 mmHg (normal range, 35–48 mmHg), an oxygen saturation of 94% (normal range, 95%-98%), and a bicarbonate of 25.8 mmol/L (normal range,18–23 mmol/L). Despite the administration of anti-infective, sputum, and asthma treatments, the symptoms did not improve. A chest CT scan revealed that the tumor in the lower lobe of the left lung was slightly smaller than before (**Figures 3B1-B2** vs **Figures 3C1-C2**). Additionally, there was bilateral lung inflammation, bronchitis, emphysema, and a small amount of pleural effusion on the left side (**Figures 3C1-C2**). In accordance with the *NCCN Guidelines for the Management of Immunotherapy-Related Toxicities, Version 1.2023*, the patient was diagnosed with immune-related pneumonia (grade 3–4) combined with pulmonary bacterial and fungal infections. Methylprednisolone sodium succinate (4mg/kg/d, commenced on day 88), micafungin sodium (100 mg q.d., from day 88 to day 92), and imipenem cilastatin sodium (1 g q.8h., from day 87 to day 98) were administered. On the following day, the patient reported a significant improvement in chest tightness, cough, and other symptoms. A CT scan revealed that the inflammation in both lungs had partially resolved after four days (**Figures 3D1-D2**) and nearly completely by the 17th day (**Figures 3F1-F2** and **Figure 4**). Thereafter, the GCs dosage was gradually reduced over a 6-week period, and the aforementioned conditions did not recur. All treatments were administered in accordance with the patient’s informed consent.

## Discussion

IrAEs are characterized by multi-system involvement, extended latency, concealment, and a proclivity for recurrence. Among these, myocarditis and pneumonia are rare and severe. The incidence of myocarditis is approximately 1.14%, while grade 3–4 hepatitis is 1–4%, and pneumonia does not exceed 5% (3, 4). Even more infrequently, multiple irAEs occur. A study with a small sample size indicated that the incidence of single irAE was 24% (148/623), and that of multiple irAEs was 9.3% (58/623) among patients receiving ICIs monotherapy, with the most common combinations being pneumonia + thyroiditis (n=7, 14%), hepatitis + thyroiditis (n=5, 10%), dermatitis + pneumonia (n=5, 10%), and dermatitis + thyroiditis (n=4, 8%). In contrast, the incidence of multiple irAEs in patients treated with a combination of chemotherapy was only 1.7% (1/33) (5).

Following two cycles of chemotherapy in combination with ICIs, the patient developed myasthenia gravis (irAEs grade 3), myocarditis (irAEs grade 4), and hepatitis (irAEs grade 3). However, the indicators of myocardial injury and liver enzyme levels returned to normal after GCs treatment (**Figures 4A, B**). Unfortunately, during the GCs taper, the patient developed pneumonia (irAEs grade 3–4, as shown in **Figures 3C1-C2**). The condition experienced a substantial improvement following the administration of high-dose GCs, immunoglobulin, and antibiotics (**Figures 3D1-F2**).

ICIs-mediated multiorgan injury may be associated with PD-L1 expression, blood neutrophile-to-lymphocyte ratio (NLR), treatment modality, and type of ICIs. In a study of 894 patients with stage IV NSCLC treated with PD-1/PD-L1 inhibitors, Daniello et al. (6) found that the occurrence of irAEs was significantly correlated with high PD-L1 expression (P = 0.003). And patients with NLR < 5 in the blood were more likely to develop irAEs (*P* < 0.001). This conclusion has also been confirmed in the findings of the study conducted by Kichenadasse et al. (7). A study by Wang et al. (8) analyzed 6270 patients with advanced NSCLC who received ICIs as monotherapy or in combination with chemotherapy. The result indicated that the incidence of grade 3/4 irAEs was lower in the combination therapy group than in the monotherapy group (7.1% vs. 10.6%, 95% CI: 0.291–0.916). Khoja et al. (9) conducted a meta-analysis of 48 clinical studies on the use of ICIs, and discovered that grade 3/4 irAEs were more common in patients treated with CTLA-4 monoclonal antibody than in those treated with PD-1 monoclonal antibody (31% vs 10%, 95% CI: 3.5–4.6).

The precise mechanisms of irAEs are not yet completely clear. The prevailing hypothesis is that ICIs reactivate T cells, resulting in the release of a high number of inflammatory factors and cytokines, which in turn causes autoimmune damage. The most serious of these is known as cytokine release syndrome (CRS) (10). Several studies have demonstrated significant differences in the blood levels of IL-1, IL-2, IL-6, IL-10, IL-13, and IFN before and after the onset of irAEs, suggesting that the cytokines are related to the occurrence of irAEs (11–14). Zhao et al. (12) also found that high pretreatment levels of blood IL-1β (>12.4 pg/mL) and IL-2 (>7.5 pg/mL) were significantly associated with irAEs. In a study by Lim et al. (15), it was found that cytokines such as IL-13 were correlated with severe irAEs, and a scoring model was developed to predict severe irAEs. Furthermore, it has also been reported that baseline IL-17 levels are associated with the occurrence of grade 3 gastrointestinal adverse events in patients treated with ipilimumab (P < 0.05) (14). Additionally, a high level of IL-6 (≥ 11.81 pg/mL) in patients undergoing treatment with ICIs has been found to be significantly correlated with the severity of immune-related pneumonia (13). In this patient, plasma IL-6 increased significantly at the outset of myocarditis, hepatitis, and pneumonia, while IL-10 increased exclusively at the onset of pneumonia, suggesting that both were associated with the development and regression of irAEs.

IL-6 is an inflammatory cytokine that exerts a broad effect on the systemic immune system and is associated with various diseases, including cancer (16). It is involved in cell survival, growth, immune regulation, and inflammation via the JAK/STAT signaling pathway. Furthermore, IL-6 signaling plays a critical role in carcinogenesis, inhibition of antitumor immunity, and promotion of tumor dissemination in the tumor environment (17). The clinical presentation of irAEs is characterized by a systemic inflammatory response, including an increase in circulating pro-inflammatory cytokines. Several studies have reported the association and predictive value of IL-6 with irAEs (18–23). Tanaka et al. reported that an increased level of IL-6 was associated with a higher incidence of psoriasis (*P* = 0.018) in melanoma patients treated with nivolumab (18). A case report found that elevated serum IL-6 and CRP were proportional to the severity of immune-related colitis, and their decreased levels were proportional to the degree of colitis remission after receiving GCs, suggesting that IL-6 and CRP may be biomarkers for the diagnosis and prediction of irAEs (19). IL-6 was also identified as a strong indicator of severe irAEs in another study (20). Additionally, Hailemichael et al (21) found that the IL6-Th17 pathway is a major contributor to immune-related enterocolitis. The IL-6 blockade therapy may be of considerable clinical value due to its important role in irAEs, and treatment of irAEs with anti-IL-6 has yielded positive outcomes in certain studies (22, 23). In conclusion, IL-6 is a highly effective biomarker for the diagnosis and prediction of irAEs, as shown in our case.

Additionally, similar to our case, the case report of Hu et al. (24) showed that IL-6 and IL-10 were both highly expressed in the patient who developed immune pneumonia, which suggests that the simultaneous increase of plasma IL-6 and IL-10 may also be helpful in the prediction of pneumonia.

Targeted anti-inflammatory therapy has the potential to control irAEs. The current main cytokine inhibitors are infliximab, adalimumab, etanercept, tocilizumab, and secukinumab (11). A class of monoclonal antibodies against TNF-α, including infliximab, adalimumab and etanercept, are able to treat irAEs. Infliximab is currently recommended for the treatment of a wide range of grade 3 and above GCs-tolerant irAEs (25), with an efficacy of 87% in immune colitis (26); adalimumab and etanercept are currently recommended for use in combination with or in addition to GCs for the treatment of severe irAEs for which GCs alone are ineffective (27). Tocilizumab, a monoclonal antibody directed against IL-6 and its receptor, has a 69% response rate in the treatment of severe or fatal CRS (23). Tocilizumab is currently approved for the treatment of CRS in people aged two years and older (28). Secukinumab is an anti-IL-17A monoclonal antibody and case reports suggest that it has significant therapeutic potential in CRS (29). In addition, ustekinumab inhibits the transformation of naïve T cells into Th cells by antagonizing IL-12 and IL-23 and inhibiting IFN-γ signaling (30). Ustekinumab has been reported to treat grade 3–4 refractory immune colitis by inhibiting IFN-γ (31). Currently, cytokine antibodies have emerged as an important treatment for irAEs. Their role in the treatment of irAEs will become increasingly important as the mechanisms of irAEs continue to be elucidated.

It is evident that GCs remain the cornerstone of first-line treatment for irAEs, offering several advantages, including rapid onset of action, ease of use, and affordability. GCs are the primary treatment for the majority of grade 2 and higher irAEs, except for hypothyroidism and other endocrine irAEs, which are treated with appropriate hormone replacement (25). The efficacy rate of high-dose GCs therapy for myocarditis is about 83.3% (15/18) (32). The rate of complete recovery from meningitis was 87.5% with GCs treatment (33); in pneumonia, the efficacy rate of GCs is as high as 91.1% (34). Numerous studies have shown that the early use and appropriate dosing of GCs are key to the successful treatment of irAEs (35). Current guidelines recommend that the dose of GCs should be increased as the grade of irAEs increases, and severe irAEs typically require high-dose GCs pulse therapy, which is then tapered as symptoms alleviate (25). A better prognosis results from early detection, timely diagnosis, and early initiation of GCs (36). Recurrence of irAEs is likely if the duration of GCs is insufficient, particularly if the tapering time is insufficient (37). Nevertheless, the organism may not benefit from a longer duration of GCs, and it is generally recommended to complete the tapering process within 4–6 weeks (27). It’s important to note that if symptoms don’t improve or worsen within 2–3 days of GCs treatment, indicating corticosteroid-resistance, the addition of biologic immunomodulators (such as immunoglobulin, mycophenolate mofetil, and others) is usually necessary (38).

Furthermore, some severe irAEs often have their risk factors. For example, the potential risk factors for myocarditis and pneumonia include individual general conditions (autoimmune disease history, age ≥ 65 years, body mass index ≥ 25 kg/m^2^, ECOG PS ≥ 2), genetic factors (genetic polymorphisms of CTLA-4, PD-1, or PD-L1), treatment-related factors (high dose of ICIs, combined treatment regimen of ICIs), tumor factors (high expression of PD-L1), and others. Additionally, the presence of cardiopulmonary basic diseases is also a significant consideration (39–42). Monitoring irAEs is equally critical as evaluating efficacy after the initiation of ICIs, and the frequency of monitoring should be increased in high-risk patients. Furthermore, immune-related myocarditis usually coexists with myositis, encephalitis, and hepatitis (43). Due to the high mortality rate of severe myocarditis (44), we routinely monitor cardiac enzymes, electrocardiograms, and cardiac function tests before each cycle. In reference to the above criteria, our patient is classified as belonging to the high-risk group of irAEs. However, this does not constitute an absolute contraindication to ICIs treatment (38). Following a comprehensive evaluation and provision of information, we chose ICIs combination chemotherapy regimen, which resulted in a PR after two cycles. We closely monitored the patient’s condition upon admission and instructed the patient in self-monitoring, which enabled us to promptly identify and effectively address the occurrence of two severe irAEs.

## Data availability statement

The original contributions presented in the study are included in the article/supplementary material. Further inquiries can be directed to the corresponding authors.

## Ethics statement

The studies involving humans were approved by Clinical Research Ethics Committee of Renmin Hospital of Wuhan University. The studies were conducted in accordance with the local legislation and institutional requirements. The participants provided their written informed consent to participate in this study. Written informed consent was obtained from the individual(s) for the publication of any potentially identifiable images or data included in this article.

## Author contributions

JX: Writing – original draft, Data curation, Investigation. YW: Data curation, Writing – original draft. MX: Data curation, Writing – original draft. DY: Writing – review & editing, Conceptualization. YG: Writing – review & editing, Data curation. DT: Writing – review & editing, Methodology. XZ: Writing – review & editing, Methodology. JC: Writing – review & editing, Methodology. QL: Writing – review & editing, Conceptualization, Supervision. YY: Writing – review & editing, Project administration, Supervision, Writing – original draft, Conceptualization, Funding acquisition, Resources.
